# Loss of Sult1a1 reduces body weight and increases browning of white adipose tissue

**DOI:** 10.3389/fendo.2024.1448107

**Published:** 2024-12-04

**Authors:** Margherita Springer, Emmanuelle Meugnier, Katharina Schnabl, Kevin Sebastiaan Hof, Marie-France Champy, Tania Sorg, Benoit Petit-Demoulière, Natacha Germain, Bogdan Galusca, Bruno Estour, Hubert Vidal, Martin Klingenspor, Jörg Hager

**Affiliations:** ^1^ Société des Produits Nestlé S.A., Nestlé Institute of Health Sciences, Lausanne, Switzerland; ^2^ Chair for Molecular Nutritional Medicine, Technical University of Munich (TUM) School of Life Sciences, Technical University of Munich, Freising, Germany; ^3^ Univ-Lyon, CarMeN Laboratory, Inserm U1060, INRAE U1397, Université Claude Bernard Lyon 1, Institut National des Sciences Appliquées de Lyon (INSA Lyon), Oullins, France; ^4^ Else Kröner Fresenius Zentrum (EKFZ) für Ernährungsmedizin, Technical University of Munich, Freising, Germany; ^5^ French National Infrastructure for Mouse Phenogenomics (PHENOMIN)-Institut Clinique de la Souris, Creation, Breeding, Phenotyping, Distribution and Archiving of Model Organisms (CELPHEDIA), National Centre for Scientific Research (CNRS), National Institute of Health and Medical Research (INSERM), Université de Strasbourg, Illkirch-Grafenstaden, France; ^6^ Maven Health, Zürich, Switzerland; ^7^ Division of Endocrinology, Centre Hospitalier Universitaire de Saint-Étienne, Saint-Etienne, France; ^8^ TAPE (Eating Disorders, Addictions & Extreme Bodyweight) Research Group, University Jean Monnet, Saint Etienne, France

**Keywords:** sulfotransferase 1A1, white adipose tissue, browning, leanness, obesity

## Abstract

**Background and objective:**

Overweight and obesity affects millions of individuals worldwide and consequently represents a major public health concern. Individuals living with overweight and obesity have difficulty maintaining a low body weight due to known physiological mechanisms which prevent further weight loss and drive weight regain. In contrast, mechanisms which promote low body weight maintenance receive less attention and are largely unknown. To uncover these intrinsic mechanisms, we investigated a human cohort of constitutionally thin (CT) individuals which maintain a low body weight and are resistant to weight gain despite exposure to an obesogenic environment.

**Methods:**

To identify novel genes that contribute to low body weight maintenance, we performed transcriptomics on adipose tissue biopsies collected from CT and normal body weight (NBW) individuals and identified sulfotransferase 1A1 (SULT1A1) as a target for further investigation in mice. Sult1a1 knockout (KO) mice were fed a standard diet to assess the impact of Sult1a1 deletion on metabolic traits. To determine if high-fat feeding recapitulated the CT weight gain resistance phenotype, Sult1a1 KO mice were fed a high-fat diet for 13-weeks. A subset of wild-type and Sult1a1 KO mice from the standard diet were further analyzed for characterization of adipose tissue respiratory capacity.

**Results:**

In comparison to NBW controls, adipose tissue from CT individuals expresses less SULT1A1. Sult1a1 KO mice weigh 10% less at the end of the study period and on a high-fat diet, Sult1a1 KO mice tended to gain less weight and had reduced fat mass at 14-weeks of age. These changes were associated with reduced fasting insulin and lessened adipose tissue inflammation and fibrosis. Subcutaneous adipose tissue from Sult1a1 KO mice on a standard chow diet had elevated leak respiration, uncoupling protein 1 (UCP1) expression and increased expression of a mitochondrial marker, VDAC, associating Sult1a1 deletion to adipose tissue browning.

**Conclusions:**

Our results associate Sult1a1 deletion with a tendency for lower body weight through remodeling of white adipose tissue towards a brown phenotype. The presence of UCP1, the expression of an additional mitochondrial protein and increased respiratory capacity suggest browning of the subcutaneous adipose tissue depot of Sult1a1 KO mice.

## 1 Introduction

Obesity is a public health issue that raises healthcare costs, decreases quality of life and increases the risk of developing a chronic illness like type 2 diabetes, cardiovascular disease and cancer (1). The coronavirus disease 2019 (COVID-19) pandemic emphasized the risks associated with carrying excess body weight — overweight and obese individuals infected with severe acute respiratory syndrome coronavirus 2 (SARS-CoV-2) have poor prognosis and elevated COVID-19 mortality (2). Obesity treatment aims to reduce body weight by creating an energy deficit through lifestyle modifications (reduced caloric intake and increased physical activity) and when indicated, via pharmacotherapy or bariatric surgery (1). With current treatments, only a fraction of individuals living with overweight and obesity are able to achieve a sustained reduction in body weight over the long-term (3–5) due to known hormonal, metabolic and neurochemical adaptations that counteract current treatments (6). While these approaches can be effective in the short and medium term, the results are transient and long-term effects are often limited. In contrast, mechanisms which promote low body weight maintenance and resistance to weight gain remain unclear and have been until recently overlooked. Certain individuals, appear to have a defense against weight gain, despite exposure to an obesogenic environment (7). This study aims to uncover potential mechanisms of intrinsic resistance to weight gain, with the objective of identifying factors that help maintain a lower body weight despite exposure to environmental pressures.

To uncover intrinsic mechanisms of low body weight maintenance, we investigated the adipose tissue of individuals with constitutional thinness (CT). We and others previously demonstrated that individuals with CT maintain a low body weight and are resistant to weight gain despite exposure to an obesogenic environment (7). Aside from their low body weight, CT individuals are similar to normal body weight (NBW) controls in terms of food intake and clinical bio-chemical parameters (7). In the past, CT individuals were often mistaken for individuals affected by anorexia nervosa (AN) due to their low body weight (8, 9). However, the CT phenotype is distinct from AN as CT individuals lack the psychiatric (no behavioral disturbances in eating and weight management) and endocrine (normal serum levels of sex and thyroid hormones) features of AN (8, 9). Additionally, CT individuals express a strong desire to gain weight and often seek advice from healthcare professionals for that purpose (8, 9).

Brown adipose tissue (BAT) is present in both rodents and humans and can suppress weight gain through activation of brown adipocytes. In contrast to white adipocytes, brown adipocytes are rich in mitochondria and express uncoupling protein 1 (*UCP1*). Brown-like adipocytes, known as brite or beige adipocytes, also express *UCP1* and reside within white adipose tissue (WAT). The induction of beige adipocytes in WAT is a process known as WAT browning. BAT activation and WAT browning have been linked to leanness in mouse models and in humans (10). On a four-week fat overfeeding protocol, CT individuals maintained a low body weight through an increase in resting energy expenditure (REE) which the investigators speculated may be linked to increased BAT activity (7). Though BAT activity was not quantified in the overfeeding study, the investigators cited a previous study in an Italian cohort which observed increased BAT activity in constitutive lean individuals (11). No evidence of adipose tissue browning was found in a recent molecular characterization comparing adipose tissue from CT and NBW individuals (12). Although previous studies with CT individuals show that energy regulation seems to play a role in the persistent low weight of CT individuals the molecular mechanisms underlying these differences are poorly described.

Molecular characterization of muscle and adipose tissue collected from CT and NBW individuals showed no significantly differentially expressed genes between muscle biopsies collected from CT and NBW individuals (12). The study also investigated mitochondrial respiratory capacity of muscle biopsies and did not detect differences between the groups (12). In contrast, molecular characterization of adipose tissue collected from CT and NBW individuals found key differences in the transcriptomic profile, morphology and function (12), pointing to the adipose tissue as the potential main contributor to the CT phenotype.

Here we report the results from an adipose tissue study comparing the transcriptome of CT individuals and NBW individuals. We show that the most downregulated transcript in the adipose tissue of CT individuals is *SULT1A1*. Using a full body *Sult1a1* KO (knockout) mouse model we show that *Sult1a1* KO results in a lean, weight gain resistant phenotype that is accompanied by subcutaneous adipose tissue browning, reduced fasting insulin and protection from tissue inflammation.

## 2 Materials and methods

### 2.1 Adipose tissue transcriptomics of human CT and NBW individuals

The local research and ethics committee of Saint-Etienne, France approved the study and all subjects gave written informed consent prior to inclusion in the study. This study was registered at clinicaltrial.gov as NCT01224561. The body mass index (BMI) of CT and NBW individuals investigated is reported as mean ± SEM. Human abdominal subcutaneous adipose tissue biopsies were collected from CT females (n = 8) and NBW females (n = 8) using a needle under local anesthesia (1% lidocaine). Fat samples were immediately frozen in liquid nitrogen and stored at -80°C. Total RNA was isolated using *mir*Vana miRNA Isolation Kit (Ambion, Life Technologies, Saint Aubin, France) and was further purified using the RNeasy kit (QIAGEN, Courtaboeuf, France). Quantity analysis was performed with a ND1000 (Ozyme, St-Cyr l’Ecole, France) and quality was assessed with a Bioanalyzer (Agilent, Les Ulis, France). 100 ng of total RNA were submitted to the GeneChip3’ IVT Express Kit (Affymetrix, Inc., Santa Clara, CA) for sample processing and chip hybridization according to the manufacturer’s instructions. Affymetrix Human HG U133 Plus 2.0 arrays, covering 47,401 transcripts were scanned with GeneChip Scanner 3000 7G (Affymetrix, Inc.). Two CT and two NBW samples were excluded due to low RNA quality or low quality Affymetrix data, as a result only 6 CT and 6 controls were analyzed. Expression values and absent/present calls were calculated using MAS 5.0 and Robust Multi-array Analysis expression summary algorithms, implemented in R (version 2.9.2) within the Affy package. Data was filtered on detection calls so that probe sets considered “absent” across all subjects. All controls probesets were also removed. Statistical analysis was performed on 35,671 probe sets with the Limma package. Probe sets with a q-value < 0.05 (adjusted p-values using an optimized FDR approach) were considered as differentially expressed. The dataset is available from the Gene Expression Omnibus database (GSE167231).

For qPCR validation of differentially expressed genes, first-strand cDNA was synthesized from 250 ng total RNA in the presence of 100 U Superscript II (Life Technologies) and a mixture of random hexamers and oligo(dT) primers (Promega, Charbonnieres-les-Bains, France). Real-time quantitative PCR assays were performed with a Rotor-Gene 6000 (QIAGEN) using a standard curve. Values were normalized using hypoxanthine guanine phosphoribosyl transferase. The primer sequences are available upon request (emmanuelle.meugnier@univ-lyon1.fr).

### 2.2 Animal studies

Phenotyping of *Sult1a1* KO mice received ethical approval from the animal studies committee at the Nestlé Institute of Health Science and the French Ministry of Research under authorization number 18574. The animal study was performed in compliance with the European Community regulation for laboratory animal care and use (Directive 2010/63/UE). Caretakers completed a weekly welfare scoresheet for each mouse to monitor health status for inclusion in the study. Sult1a1 KO mice were developed as described in a previous publication (13). This animal study had four experimental groups: wild-type (WT) mice on a standard diet, *Sult1a1* KO mice on a standard diet, WT mice on a high-fat diet (HFD) and *Sult1a1* KO mice on a HFD with a sample size of n = 15 per experimental group (totaling 60 mice in this animal study). HFD feeding was conducted on weight-matched WT and Sult1a1 KO mice to standardize baseline weights between genotypes and eliminate potential confounders such as litter size and housing density, which can impact body weight. At six-weeks old, male mice were removed from their home cages and moved to experimental cages in groups of 2-4 mice per cage and left to acclimate for one week. At 7 weeks old, the chow diet (D04, Safe) was removed and replaced with either a standard diet (D12450H, Research Diets) with 10% kcal from fat or a HFD (D12451i, Research Diets) with 45% kcal from fat for 14 weeks. All mice had ad libitum access to food and water and were maintained on a 12-hour light/dark cycle at a temperature between 20-24°C and a relative humidity between 50-60%. Six mice died over the study: four WT on a standard diet, one Sult1a1 KO fed on standard diet and one WT fed with a high fat diet. Data collected up until death was included in the study. Body weight was recorded at the same time each week for the body weight tracking experiment.

### 2.3 Indirect calorimetry

Oxygen (VO_2_) consumption, carbon dioxide (VCO_2_) production, activity and food intake were measured using the LabMaster system (LabMaster, TSE Systems). Mice aged 14-weeks old were individually housed in the LabMaster system cages for a total of 48 hours under a 12h light/12h dark photoperiod at ambient temperature (21°C ± 2). The first 24 hours served to acclimatize the mice while the last 24 hours of recording were used for analysis. The data pertaining to the 24-hour recording was handled by a custom script in Python (Library used: Pandas). Measurement intervals during the experiment were at 10-minute intervals. We accordingly labelled each 10-minute interval to their consecutive hour and then aggregated the data on each hour interval and the individual mouse box. Using the aggregation, we calculated the mean, resulting in VO_2_(hourly) and VCO_2_(hourly) values. This output was used to substitute in the following equation EE (J) = 15.818(VO_2_(hourly)) + 5.176(VCO_2_(hourly)) previously described (13).

### 2.4 Body composition measurements

Total lean and fat weight were measured using quantitative Nuclear Magnetic Resonance (qNMR) (Minispec, Bruker) during the light period on conscious fed mice prior to indirect calorimetry. Body composition was measured a second time using qNMR on 20-week old mice.

### 2.5 Intraperitoneal glucose tolerance test and insulin measurement

IPGTT was performed on 16-weeks old mice that were fed either a standard diet or a HFD for 10 weeks. In parallel, blood insulin was measured during the IPGTT. Prior to glucose injection, blood was collected from the tail vein to measure fasting glucose and fasting insulin. After blood was collected for fasting measurements, the mice were administered glucose at a dose of 2 g/kg body weight by intraperitoneal injection. For blood glucose measurements, blood was collected at 15, 30, 60, 90 120,150 and 180 minutes after injection and measured using glucose monitor and test strips (Roche Diagnostics, Accutrend). Insulin during the IPGTT was measured 15 and 30 minutes after the glucose injection using 20 µl of blood. Insulin levels were measured on a BioPlex analyser (BioRad) using the Mouse Metabolic Magnetic Bead Panel Kit (Cat # MMHMAG-44K, Millipore). The IPGTT and insulin measurement were performed after an overnight fast (16-hours) during the light period.

### 2.6 Intraperitoneal insulin tolerance test

IPITT was performed on mice 18-weeks old that were fed either a standard diet or a HFD for 12 weeks. Blood collected from the tail vein was used to measure fasting glucose prior to insulin dosing. Insulin was administered by intraperitoneal injection with a standardized insulin load of 1 IU/kg body weight. Blood glucose was measured at 15, 30, 60, 90 and 120 minutes post insulin injection using a blood glucose monitor and glucose test strips (Roche Diagnostics, Accutrend). The test was conducted during the light period after 4 hours fasting.

### 2.7 Adipose tissue histology

The right epididymal white adipose tissue (eWAT) was collected, weighted and then fixed in formalin (Leica), paraffin embedded and dehydrated in a gradient of 70, 95 and 100% ethanol. The tissue was incubated in Sub-X (Leica) before embedding in paraplast (Leica). The eWAT blocks were sectioned 7 μm thick with three sections per slide. Slides were stained with hematoxylin and eosin (H & E) and Sirius Red (SR) according to standardized protocols of the EPFL Histology Platform (14). Images were acquired using a slide scanner (VS-120, Olympus) and scale bars were added using Fiji (ImageJ) software. The H & E stained images were uploaded to a publicly available bioimaging software, QuPath (https://qupath.github.io/), and the total number of nuclei were counted and then adjusted to the total area of the sample.

### 2.8 Respirometry of adipocytes

#### 2.8.1 Isolation and preparation of adipocytes

Oxygen consumption of adipocytes was measured according to previously established methods (15, 16) in 29-week-old mice on a standard diet. Adipocytes from the inguinal white adipose tissue (iWAT) and eWAT of WT (n = 6) and Sult1a1 KO (n = 4) were separated from other cell types by collagenase digestion (1 g/L Collagenase B (Cat # 11088815001, Sigma) in Hank’s Balanced Salt Solution (Cat # 14025050, Thermo Fisher) supplemented with 4% BSA) according to a method previously described (15, 16). Adipocytes were washed with STE buffer (250 mM sucrose (Carl Roth), 5 mM Tris (Carl Roth), 2 mM EGTA (Carl Roth), pH = 7.4 at 4°C) containing 4% BSA-fatty acid free and kept on ice until measurement.

#### 2.8.2 High-resolution respirometry using Oroboros

Oxygen consumption of isolated adipocytes was measured using high-resolution respirometry (Oxygraph-2k, OROBOROS INSTRUMENTS, Innsbruck, Austria) previously described (15, 16). Approximately 100 μl isolated adipocytes were added to the chamber containing oxygenated MIR05 buffer (110 mM sucrose, 60 mM potassium lactobionate, 0.5 mM EGTA, 3 mM MgCl_2_*6H_2_O, 20 mM taurine, 10 mM KH_2_PO_4_, 20 mM Hepes, 1 g/L BSA-fatty acid free and 5 mM malate, pH 7.1 described by OROBOROS INSTRUMENTS). To measure basal respiration 5 mM pyruvate and 5µM malate were added to the chamber. Leak respiration was measured by inhibiting ATP synthase with 2 µg/ml oligomycin. Maximal respiration was measured using a FCCP titration (in 0.5 µM steps). Non-mitochondrial respiration was measured by adding 2.5 µM antimycin A and subtracted from all other respiratory states. After measurement, the adipocyte suspension was collected to quantify DNA concentration. Samples were kept at -80°C and then thawed on ice prior to DNA extraction.

### 2.9 DNA extraction and quantification

Genomic DNA (gDNA) was isolated from 500 µl of adipocyte suspension with the DNeasy Blood and Tissue Kit (Cat # 69504, Qiagen) according to the manufacturer’s recommendations with the following modification to accommodate the larger volume of sample needed for DNA extraction. After adding ethanol and mixing for 30 seconds, 600 µl of mixture was added to a spin column and centrifuged for 1 minute at 8000 rpm. The flow-through was discarded and then the spin column was added to a fresh tube and then the remaining volume of sample was added to the spin column and then centrifuged for 1 minute at 8000 rpm. The wash steps and elution were performed according to the protocol provided by Qiagen. DNA content of the adipocyte suspensions was quantified by quantitative PCR amplifying the resistin promoter with the primer sequences described previously (15, 16). gDNA content of the adipocyte suspension was estimated from a standard curve of known gDNA concentrations.

### 2.10 Western blot

Protein was extracted from isolated adipocytes with sucrose lysis buffer (10 ml M-PER (Cat # 78501, Thermo Scientific), 250 nM sucrose (Cat # 84100, Sigma Aldrich), 1% NP-40 (Cat # 492016, Calbiochem), 1mM EDTA (Cat # AM9260G, Ambion) and protease & phosphatase inhibitors (Cat # A32961, Thermo Scientfic). Protein concentration of the adipocytes was quantified using the DC Protein Assay kit according to the manufacturer’s instructions (Cat # 5000116, Bio-Rad). 15 μg of adipocyte protein lysate was separated on a 4-12% Bis-Tris gel (Cat # NW04127, Invitrogen) and transferred to a nitrocellulose membrane (Cat # 1704158, Bio-Rad). The nitrocellulose membrane was incubated overnight with antibodies targeting UCP1 (Cat # 14670, Cell Signaling), VDAC (Cat # 4661, Cell Signaling) and vinculin (Cat # 13901, Cell Signaling). The dilutions used for anti-UCP1, anti-vinculin and anti-VDAC were 1:500, 1:1000 and 1:1000, respectively. Western blots were quantified using ImageJ, a publicly available software available at https://imagej.net/ij/.

### 2.11 Statistical analysis

Differential gene expression analysis from the human subjects was performed in R with the Affy and Limma packages and qPCR data from the human subjects was analyzed in GraphPad Prism 8 software (GraphPad Software, San Diego, California, USA) and p values were calculated using an unpaired Student’s t test.

Data from the animal studies were analyzed in GraphPad Prism. P values lower than 0.05 were considered statistically significant (shown on plots as p < 0.05 = *, p < 0.01 = **, p < 0.001 **, p < 0.0001 = **** and for trends the calculated p-value is shown). For pairwise comparisons, we have tested the normal distribution using the Shapiro-Wilk test and the F test to check for equality of variances. We used a parametric test (t-test) when the Shapiro-Wilk and F statistic were not statistically significant p > 0.05 and we performed nonparametric tests (Mann-Whitney test) when the Shapiro-Wilk and F statistic were statistically significant (p < 0.05). For the animal experiments with repeated measures a two-way ANOVA mixed-effects analysis was used with Sidak’s correction for multiple comparisons. A linear regression for energy expenditure (EE) and body weight was performed in R Studio to check homogeneity of the regression slopes. If the F statistic was not statistically significant (p > 0.05) homogeneity of the regression slopes was assumed. Normality of residuals and homogeneity of residual variances were assumed if the Shapiro-Wilk and Levene’s test were not statistically significant (p > 0.05). A two-way ANCOVA with Bonferroni correction for multiple testing was used to compare the effect of genotype at each hour of EE recording with body weight as a covariate.

## 3 Results

### 3.1 Reduced expression of SULT1A1 in the adipose tissue of the constitutionally thin

**Figure 1:**
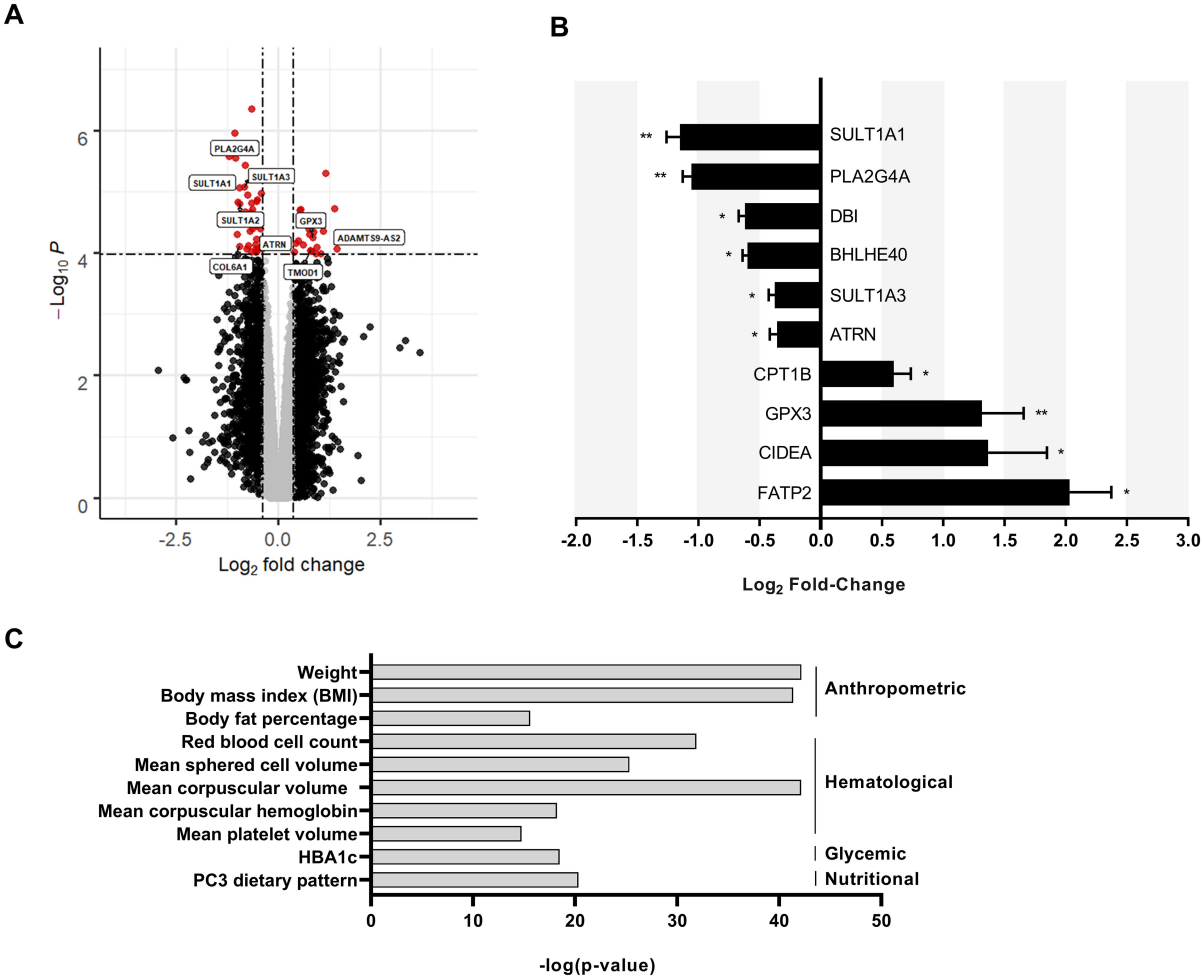
Reduced expression of SULT1A1 in the adipose tissue of CT individuals. **(A)** Volcano plot enabling visualization of differentially expressed probesets with a q-value<0.05 between CT (n =6) and normal body weight individuals (n=6). Dashed lines help to visualize the thresholds corresponding to our statistical analysis and represent a log_2_ fold change (≥|0.38|) and a -log_10_ p-values ≥ 1.05.10^-4^. Grey dots represent transcripts that are not significantly differentially expressed. Black dots are transcripts that are differentially expressed by not statistically significant. Red dots are transcripts that are differentially expressed and statistically significant. **(B)** Expression of selected differentially expressed transcripts by qPCR from RNA collected from CT and normal body weight individuals shown in **Figure 1**, **(A)** p values were calculated using an unpaired Student’s t test *p ≤ 0.05 and **p ≤ 0.01 **(C)** Data obtained from the Common Metabolic Disease Knowledge Portal associating SULT1A1 variants with metabolic phenotypes. Top most significant associations are shown and grouped by type.

Subcutaneous adipose tissue from the CT (BMI = 17.1 ± 0.3 kg/m^2^) and NBW individuals (BMI = 21.7 ± 0.4 kg/m^2^) were compared to identify differentially expressed genes by microarray. After filtering, 35,671 probesets were included in the analysis. Of the 35,671 probesets, 54 probesets corresponding to 44 known genes were significantly differentially expressed (with a q-value < 0.05) (**Figure 1A**). The most significantly differentially expressed transcripts were validated by qPCR. The most downregulated transcript in the adipose tissue of the constitutionally thin was *SULT1A1* (**Figure 1B**) which codes for a cytosolic protein involved in the transfer of sulfonate groups to endogenous and exogenous substrates within the body (17) with no described function in adipose tissue. We conducted a search of the Common Metabolic Disease Knowledge Portal (https://md.hugeamp.org/) to check the association between SULT1A1 and metabolic phenotypes and found that variants of SULT1A1 were associated with anthropometric, hematological, glycemic and nutritional traits (**Figure 1C**) (29).

### 3.2 Sult1a1 KO mice show a tendency for reduced body weight

**Figure 2:**
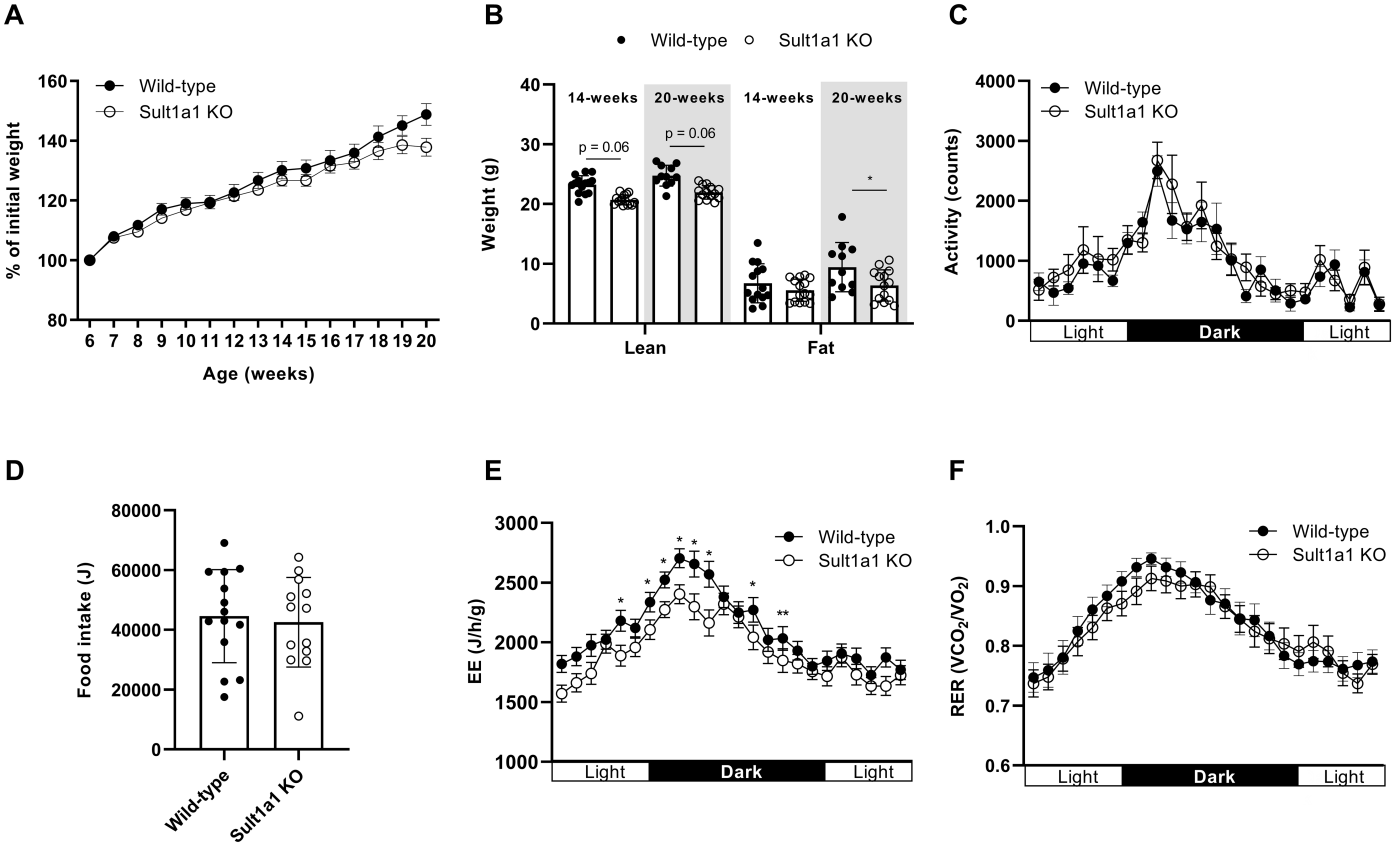
Sult1a1 KO have a tendency for reduced body weight. **(A)** Body weight of male wild-type (WT) and Sult1a1 knockout (KO) mice on a standard diet for 14-weeks (n = 11-15 mice at each time point) shown as a percentage of baseline. **(B)** Body composition of mice 14 and 20 weeks-old on a standard chow diet. **(C)** 24-hour activity of mice housed in metabolic cages at 14-weeks old. **(D)** 24-hour food intake of mice housed in metabolic cages at 14-weeks old. **(E)** EE of mice housed in metabolic cages at 14-weeks old adjusted for body weight as a covariate. EE in units J/h/g. **(F)** RER of 14-week old chow-fed mice, data represented as the mean ± SEM. A mixed effects model was used to determine effect of genotype (p > 0.05). Data represented as mean ± SD for **(B, D)**, for all other panels the mean ± SEM is shown.

As *SULT1A1* was among the most downregulated transcript in CT adipose tissue, the contribution of SULT1A1 towards CT was evaluated by characterizing the metabolic phenotype of full body *Sult1a1* KO mice. *Sult1a1* KO mice are viable and fertile and displayed no obvious macroscopic abnormalities. The body weight of male WT controls and *Sult1a1* KO mice was tracked weekly from the age of 6 to 20 weeks old mice on a standard diet. At the onset of the study, Sult1a1 KO mice weighed less than WT mice. At 13-weeks of age, Sult1a1 KO mice began to gain less weight than WT mice and at the end of the study Sult1a1 KO mice, weighed approximately 10% less than WT mice (**Figure 2A**).

On a chow diet at 14-weeks of age, Sult1a1 KO mice have a tendency for reduced lean mass (p = 0.064) but no difference in fat mass (p = 0.869). At 20-weeks of age Sult1a1 KO mice have a tendency for reduced lean (p = 0.062) and reduced fat mass (0.029) (**Figure 2B**). At 14 weeks old, food intake and activity were recorded using the TSE PhenoMaster System. Food intake over 24-hours was not significantly different between the groups. The light/dark time course plot for activity shows a tendency for Sult1a1 KO to have a higher activity towards the end of the light phase and beginning of the dark phase however a two-way ANOVA found no significant effect of genotype at each hour recorded (F (1, 26) = 1.122, p = 0.2795)) (**Figure 2C**). The total 24-hour activity (WT = 23441.01 ± 4975.53 counts vs *Sult1a1* KO = 26094.79 ± 2109 counts; p = 0.28) was also not different between the genotypes of chow fed-mice nor was food intake (WT = 44597.26 ± 15580.55 J vs *Sult1a1* KO = 42550.96 ± 14998.68 J; p = 0.731) (**Figure 2D**) indicating that changes in food intake and activity do not account for the reduced body weight of *Sult1a1* KO mice.

Energy expenditure (EE) was adjusted for body weight as a covariate using ANCOVA (linear regression for EE and body weight shown in **Supplementary Figure 1A**) (31–34). EE was lower in Sult1a1 KO mice at several time points during the light and dark phase (**Figure 2E**). When the adjusted data was analyzed by grouping total, light and dark Sult1a1 KO mice had a significant reduction in total EE (p < 0.05) and in EE during the light phase (p < 0.05) (**Supplementary Figure 1C**) in comparison to control chow-fed mice. The respiratory exchange ratio was similar between the genotypes (F (1, 13) = 0.2220, p = 0.6453)) (**Figure 2F**) and when the data was grouped into total, light and dark there was no significant difference between the genotypes (p > 0.05) (**Supplementary Figure 1D**) indicating that during indirect calorimetry energy usage was similar between both groups of chow-fed mice.

### 3.3 Sult1a1 KO mice are resistant to weight gain and protected against features of diet induced obesity

**Figure 3:**
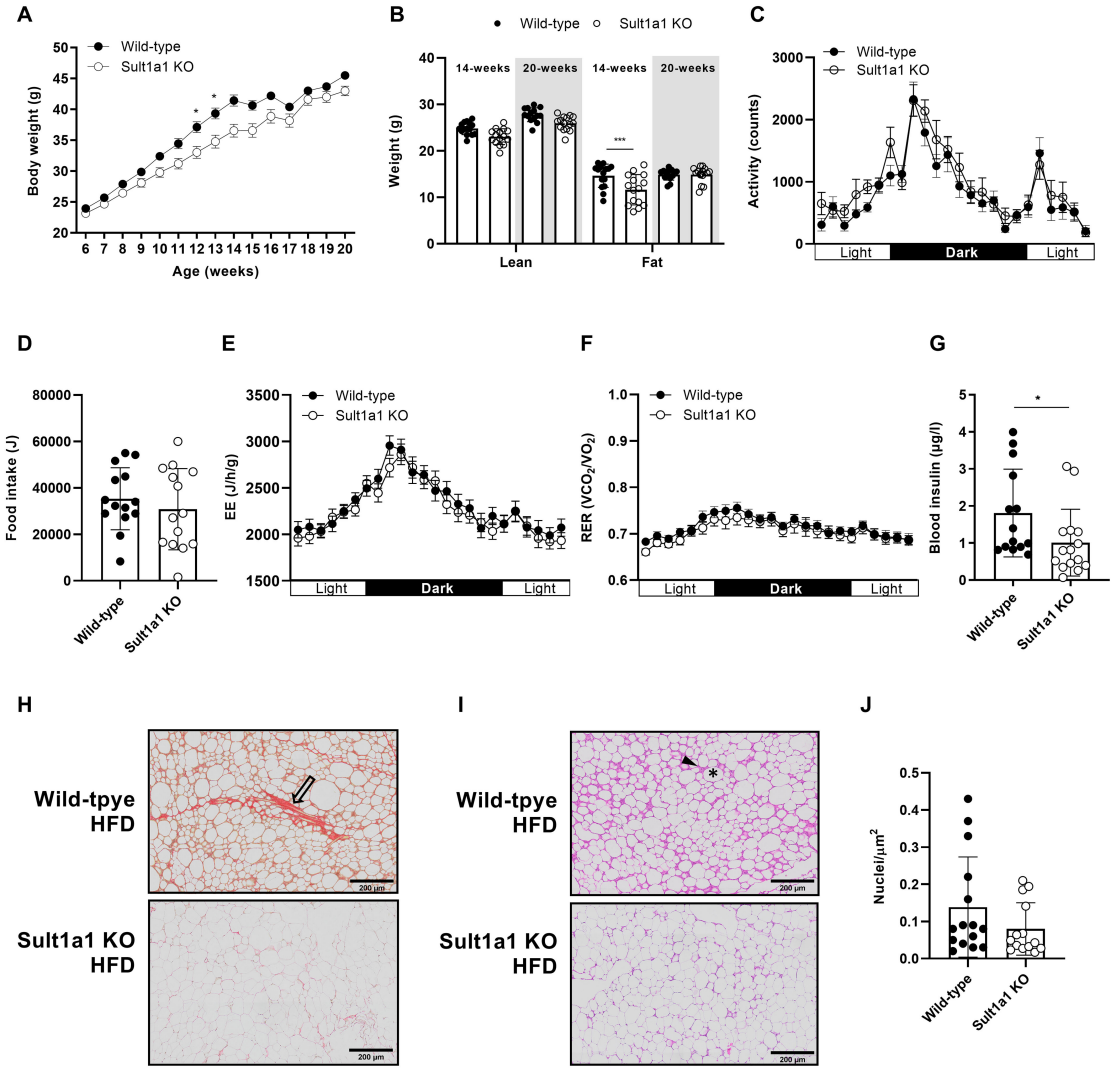
Sult1a1 KO mice are resistant to weight gain and are protected from features of diet induced obesity. **(A)** Body weight of WT and Sult1a1 KO mice on a HFD for 13-weeks (n = 14-15 mice at each time point). **(B)** Body composition of 14 and 20-week old mice fed a HFD. **(C)** Activity shown in unit counts for WT and Sult1a1 KO mice on a HFD for 13-weeks **(D)** Food intake of mice at 14-weeks old on a HFD during indirect calorimetry **(E)** EE of mice at 14-weeks old on a HFD during indirect calorimetry. Data adjusted for body weight as a covariate and shown in units J/h/g. **(F)** RER of mice 14-weeks old on a HFD during indirect calorimetry **(G)** Fasting plasma insulin levels of 16-week old WT and Sult1a1 KO mice (n = 15 per genotype). **(H)** Representative image of SR stained eWAT from WT and Sult1a1 KO mice on a HFD for 13 weeks. Open arrowheads indicate collagen deposits. **(I)** Representative image of hematoxylin and eosin (H & E) stained eWAT from WT and Sult1a1 KO mice on a HFD for 13 weeks. Arrowhead indicates immune cell infiltration and star indicates lysed adipocyte remnants. **(J)** Quantification of total nuclei in H & E stained images normalized to total area of tissue section. Data represented as mean ± SD for **(B, D, G, J)**; for all other panels mean ± SEM is shown.

Since CT individuals are resistant to weight gain even when food intake is increased beyond their energy requirement (7), it was hypothesized that *Sult1a1* KO mice would be resistant to weight gain. To test this hypothesis, a separate cohort of weight matched WT and *Sult1a1* KO mice were provided a HFD for 13-weeks (n = 14-15 per genotype). At the onset of the study, the body weight of the Sult1a1 KO and WT mice began to diverge with body weight being significantly reduced at 13 and 14 weeks of age (p < 0.05) (**Figure 3A**) with the mid-phase of the study recapitulating features of the weight resistance phenotype of CT. On a high-fat diet, mice 14-weeks old have no significant difference lean mass (p = 0.220) but a reduction in fat mass (p = 0.001). The reduction in fat mass appeared to be transient and at 20-weeks of age lean mass and fat mass is similar between the genotypes, p = 0.203 and p = 0.999, respectively (**Figure 3B**).

In the cohort of HFD-fed mice we also measured activity, food intake, EE and RER but found no differences between the genotypes (**Figures 3C–F**). When the hourly data was collected into total, light and dark to check for differences during the activity phases, there was no difference between the genotypes for EE and RER (**Supplementary Figures 1C, D**).We investigated if the resistance to weight gain observed in *Sult1a1* KO mice could provide protection against the harmful effects of diet induced obesity. At 16-weeks old, after 9 weeks on a HFD, *Sult1a1* KO mice tended to have lower fasting plasma glucose (WT = 182.4 ± 12.88 mg/dl; *Sult1a1* KO = 154.9 ± 9.63 mg/dl; p = 0.10) (**Supplementary Figure 2A**) and significantly lower fasting plasma insulin compared to WT mice (WT = 1.797 ± 0.2947 µg/l; *Sult1a1* KO = 1.011 ± 0.2321 µg/l; p < 0.05) (**Figure 3G**). A summary of metabolic characteristics of WT and Sult1a1 KO mice on chow and HFD are listed in **Table 1**.

**Table 1:** Summary of metabolic characteristics of WT and Sult1a1 KO mice on chow and HF.

Characteristic	Wild-type chow	Sult1a1 KO chow	p-value	Wild-type HFD	Sult1a1 KO chow	p-value
Body weight (14-weeks) [g]	32.53 ± 3.26	28.61 ± 2.12	0.013	41.41 ± 3.52	36.55 ± 4.08	0.024
Body weight as a percent of initial (14-weeks) [%]	130.15 ± 10.84	126.68 ± 7.31	0.997	NA	NA	NA
Body composition (lean, 14-weeks) [g]	23.26 ± 1.48	20.69 ± 0.82	0.064	24.81 ± 1.32	23.05 ± 1.69	0.220
Body composition (fat, 14-weeks) [g]	6.74 ± 3.26	5.57 ± 1.86	0.869	14.64 ± 2.51	11.62 ± 3.25	0.001
Body weight (20-weeks) [g]	37.15 ± 4.34	31.14 ± 3.00	0.017	45.47 ± 2.24	42.98 ± 2.96	0.217
Body weight as a percent of initial (20-weeks) [%]	148.77 ± 11.98	137.79 ± 11.24	0.3630	NA	NA	NA
Body composition (lean, 20-weeks) [g]	24.74 ± 1.75	21.94 ± 1.14	0.062	27.76 ± 1.50	25.93 ± 1.54	0.203
Body composition (fat, 20-weeks) [g]	9.42 ± 4.12	6.38 ± 2.61	0.029	14.89 ± 1.19	14.88 ± 1.69	0.999
Average 24-h locomotor activity (14-weeks) [counts]	23341.07 ± 4975.53	26094.79 ± 2109.00	0.278	21261.21 ± 3496.76	24748.67 ± 4151.25	0.028
Average 24-h food intake (14-weeks) [J]	44597.26 ± 15580.55	42550.96 ± 14998.68	0.731	35325.72 ± 13372.25	31999 ± 14780.78	0.452
Average 24-h Respiratory exchange ratio (14-weeks) [VO_2_/CO_2_]	0.85 ± 0.10	0.82 ± 0.09	0.836	0.71 ± 0.02	0.71 ± 0.02	0.111
Average 24-h energy expenditure (14-weeks)	2102.42 ± 295.10	1928.63 ± 247.23	0.043	2313.04 ± 281.32	2262.75 ± 277.79	0.536

NA, Not Applicable.

Area under the curve (AUC) of the IPGTT was similar between WT and Sult1a1 KO mice and was not statistically significant (AUC blood glucose WT = 52333 ± 2939; AUC blood glucose *Sult1a1* KO = 49869 ± 1967; p > 0.05) (**Supplementary Figure 2B**). Insulin output during the IPGTT, assessed by measuring basa; blood insulin concentration and at 15 and 30 minutes post glucose injection, was not statistically different between *Sult1a1* KO and WT mice (AUC blood insulin WT = 55.33 ± 14.77; AUC blood insulin *Sult1a1* KO = 55.96 ± 13.64; p > 0.05) (**Supplementary Figure 2C**). An insulin tolerance test was performed to evaluate insulin sensitivity in 18-weeks old mice after 11 weeks on a HFD. *Sult1a1* KO mice tended to have a very modest reduction in blood glucose in response to an IP injection of insulin throughout the test. AUC glucose from 0 to 120 minutes was lower in *Sult1a1* KO mice compared to WT mice but was not statistically significant (AUC blood glucose WT = 21334 ± 2247; AUC blood glucose *Sult1a1* KO = 18886 ± 1893; p = 0.41) (**Supplementary Figure 2D**).Since reduced *SULT1A1* expression was observed in CT adipose tissue, the adipose tissue of *Sult1a1* KO was further characterized. eWAT sections were stained with Sirius Red (SR) to identify fibrotic collagen deposits. In comparison to *Sult1a1* KO eWAT, sections from WT mice on a HFD appeared to have increased collagen deposition suggesting possible adipose tissue fibrosis while *Sult1a1* KO adipose tissue had fewer collagen streaks (**Figure 3H**). H&E stained sections of eWAT from WT mice had darkly stained patches which surrounded large circles while eWAT from *Sult1a1* KO mice had fewer dark patches and classical white adipocyte morphology (**Figure 3I**). Inspection of the eWAT sections confirmed that the darkly stained patches were infiltrated with immune cells and that the large symmetrical circles were lysed adipocyte remnants. The number of nuclei in the H & E stained sections were quantified to estimate the number of infiltrating immune cells (**Figure 3J**). The number of nuclei/μm^2^ tended to be higher in WT mice but was not statistically significant (p > 0.05).

### 3.4 Loss of *Sult1a1* increases leak respiration and expression of mitochondrial proteins in iWAT

The respiratory capacity of adipocytes was measured as a previous study identified an increase in the mitochondrial respiratory capacity of CT adipocytes (12). The mitochondrial respiratory capacity of WT and Sult1a1 KO adipocytes was measured using high-resolution respirometry (Oroboros). From representative subcutaneous (iWAT) and visceral (eWAT) depots, adipocytes were isolated and respiratory capacity was measured from a subset of mice on the chow diet.

**Figure 4:**
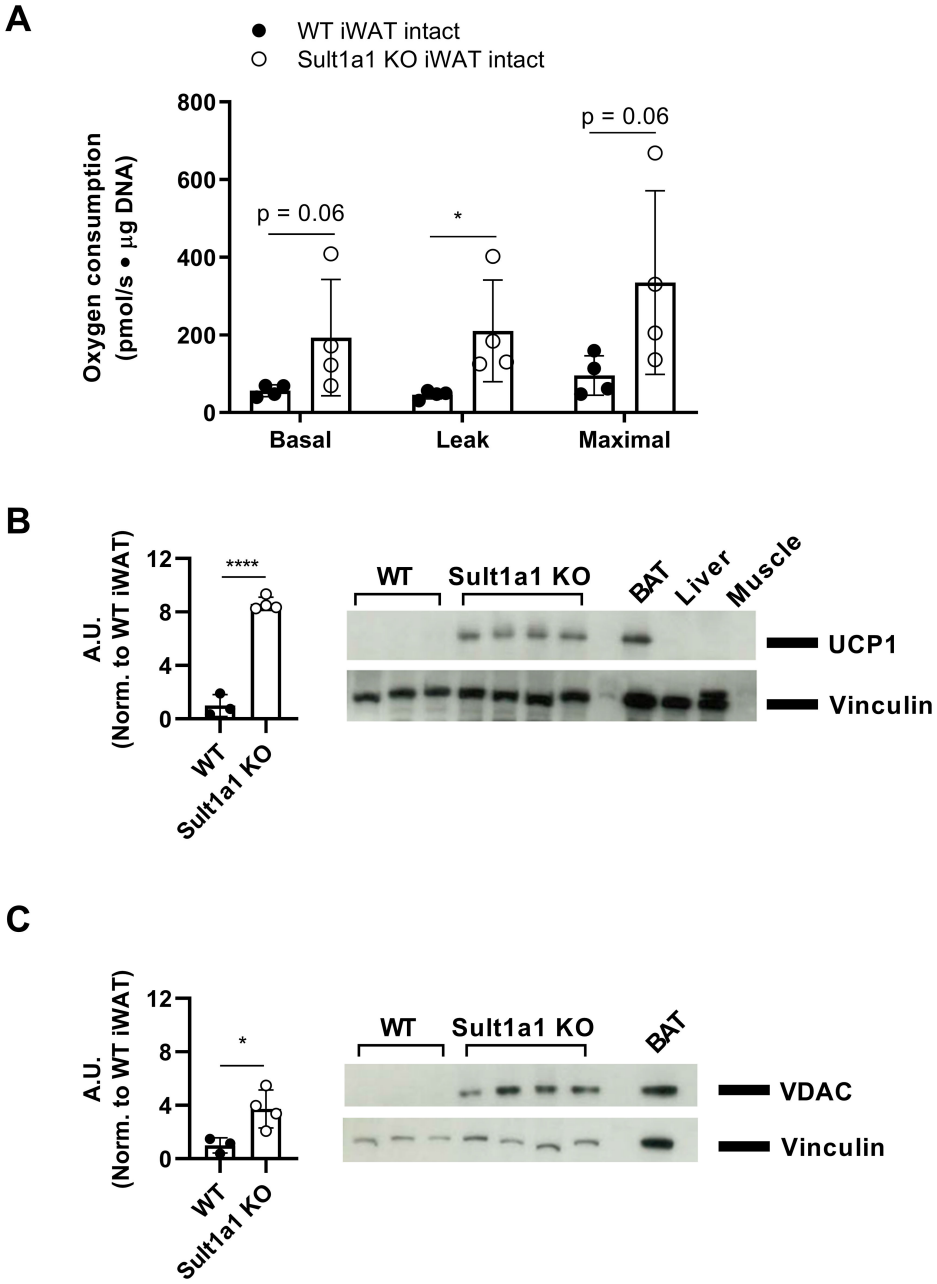
Loss of Sult1a1 increases leak respiration and expression of mitochondrial proteins in iWAT. **(A)** Higher respiratory capacity in intact subcutaneous adipocytes (iWAT) from Sult1a1 knockout mice (n = 4 mice/group). Basal respiration was measured using pyruvate (5µM) and malate (5µM) as a substrates. Leak respiration was measured by inhibiting ATP synthase with oligomycin (2µg/ml). Titration of FCCP (in 0.5 µM steps) was used to determine maximal respiration. Non-mitochondrial respiration was measured by adding antimycin A (2.5 µM) and subtracted from all other respiratory states. Oxygen consumption was normalized by µg of DNA per chamber. p values were calculated by using the Mann-Whitney test. **(B)** Quantification of western blot using anti-UCP1 antibody on protein lysates isolated from iWAT analyzed in **Figure 4A**. Vinculin serves as loading control. First three lanes are WT iWAT lysate followed by four Sult1a1 KO iWAT lysate. The last three lanes are controls; brown adipose tissue (BAT) serves as positive control and expresses UCP1 protein, liver and muscle serve as negative control and do not express UCP1. **(C)** Quantification of Western blot using anti-VDAC antibody on protein lysates isolated from iWAT analyzed in **Figure 2A**. Vinculin serves as loading control. First three lanes are WT iWAT lysate followed by four Sult1a1 KO iWAT lysate. The last lane is BAT which serves as positive control for mitochondrial protein expression. p values for both quantifications were calculated using a t-test. Data represented as mean ± SD for all panels.

Primary adipocytes isolated from iWAT showed increased basal, leak and maximal respiration (**Figure 4A**) with a statistically significant increase in leak respiration in Sult1a1 KO adipocytes (WT = 46.02 ± 10.65 pmol/s · mg DNA vs *Sult1a1* KO = 210.28 ± 130.66 pmol/s · mg DNA; p < 0.05). Since *Sult1a1* KO mice had an increase in leak respiration and leak respiration can be driven by UCP1, its expression was measured from the same adipocytes isolated from the chow-fed mice shown in **Figure 4A**. UCP1 expression was increased in the iWAT of Sult1a1 KO mice with the quantification and Western Blot shown in **Figure 4B**. Voltage-dependent anion-selective channel 1 (VDAC), a protein of the outer mitochondrial membrane, was measured and was increased in the iWAT tissue of *Sult1a1* KO mice (**Figure 4C**) suggesting increased mitochondrial content in the iWAT depot of *Sult1a1* KO mice. Intact primary adipocytes from eWAT had a higher but not statistically significant increase in basal respiration and maximal respiratory capacity (**Supplementary Figure 3**). Leak respiration in adipocytes isolated from eWAT was similar in WT and *Sult1a1* KO adipocytes suggesting that the browning phenotype is specific to the iWAT depot of *Sult1a1* KO mice. In summary, the subcutaneous adipose tissue of *Sult1a1* KO mice has increased leak respiration, increased UCP1 expression and increased mitochondrial content indicating browning of *Sult1a1* KO subcutaneous adipose tissue.

## 4 Discussion

Constitutional thinness is a phenotype of human leanness and resistance to weight gain (7). Insight into the molecular basis of constitutional thinness may provide a better understanding of conditions like obesity where there is a physiological resistance to changes in body weight and eventually may provide new targets for sustained weight maintenance. To identify novel genes that contribute to low body weight maintenance, we performed transcriptomics on adipose tissue biopsies collected from CT and normal body weight individuals and identified *SULT1A1* as a target for further investigation as it was among the most downregulated transcript in the adipose tissue of the CT individuals. The *SULT1A1* transcript codes for a cytosolic protein which catalyzes the transfer of sulfonate groups from the universal sulfur donor 3′-phosphoadenosine 5′-phosphosulfate (PAPS) to endogenous and exogenous substrates within the body (17). Substrates for SULT1A1 include molecules which contain a phenolic ring bearing a hydroxyl or amine group (17). Addition of a sulfur group, a process knowns as sulfate conjugation, increases the water solubility of molecules facilitating excretion and often lowers the biological activity of molecules (17). Sulfate conjugation is an important mechanism for detoxification and elimination of exogenous compounds (drugs, bioactive food components and pollutants). However, sulfate conjugation can also bioactivate molecules, typically ones found in processed foods, generating chemical species which can covalently bind to DNA and promote carcinogenesis (18–20). The expression and activity of SULT1A1 in tissues involved in exogenous compound metabolism (liver, gastrointestinal tract, kidney and lung) has been characterized in humans and in mice (21, 22). A role or function for SULT1A1 in adipose tissue has not been previously described. Based on the role of sulfotransferases and their tissue distribution, reduced SULT1A1 expression may impair the metabolism of small exogenous phenolic molecules such as polyphenols, which can have potential health benefits. This hypothesis has been tested in the SGBS cell line, a model for human adipocytes, where SULT1A1 silencing using siRNA led to higher intracellular concentrations of resveratrol, a polyphenol and known substrate of SULT1A1. Resveratrol’s bioavailability was enhanced due to reduced sulfonation and its metabolite, resveratrol-3’-sulfate, was present at lower intra- and extracellular levels (23). SULT1A1 does have some affinity for endogenous substrates like thyroid hormone, dopamine and estrogen however humans have SULT1B1, SULT1A3 and SULT1E1 which sulfonate the aforementioned endogenous substrates (17, 24). Previous studies which measured the plasma concentration of thyroid hormone of CT and controls found no difference between the groups (7). Future studies could investigate whether changes in polyphenol metabolism or in hormone-related pathways contribute to the phenotype observed in CT. Though genome wide association studies (GWAS) have linked susceptibility loci mapping close to *SULT1A1* with BMI (25, 26), the metabolic phenotype of *Sult1a1* KO mice has not been described. Possible reasons for the lack of *Sult1a1* loss of function studies maybe be due to previous investigations which either found through fine mapping of a lead obesity SNP (rs7359397) region no association of the *SULT1A1* variants with BMI (27) or no change (hypothalamus) and opposing (liver and adipose) patterns of *Sult1a1* mRNA expression in rats fed a HFD (28).

Through investigating the CT phenotype, we had evidence linking reduced *SULT1A1* expression in adipose tissue to human leanness and selected *SULT1A1* as a target for further investigation in mice. Metabolic traits of full body *Sult1a1* KO mice were characterized on a standard and HFD to determine if reduced *Sult1a1* contributes to the CT phenotype. The results of this study show that *Sult1a1* KO mice mirror some features of the CT phenotype associating *Sult1a1* KO to leanness and resistance to weight gain. At 13-weeks of age, the percentage of weight gain begins to reduce in the Sult1a1 KO mice on a chow diet. Body composition measurements at 14-weeks of age suggest that the reduction is due to a reduction in lean mass rather than fat mass. At 20-weeks, when there is a more apparent reduction in weight gain, the reduced body weight of the mice on a chow diet appears to be driven by a tendency for reduced lean mass and a statistically significant reduction in fat mass. In a cohort of male and female CT humans, the body composition measurements also indicated an overall reduction in lean and fat mass when adjusted for sex and for age (12).

In comparison to control mice, *Sult1a1* KO mice on a chow diet weigh 10% less whereas CT humans weigh 25% less than normal body weight controls (7). The difference in magnitude suggests that reduced *Sult1a1* expression is one of possibly many factors that contribute towards human leanness. Indeed, two recent GWAS investigating thinness indicate that like obesity, thinness is polygenic (25, 30). For example, recently the *ALK* gene was identified in a GWAS comparing an Estonian cohort of thin and control individuals and functionally linked to thinness. *ALK* variants were associated with thinness and genetic deletion of *Alk* in mice resulted in reduced body weight demonstrating a link between ALK and weight regulation (30). In this study, *Sult1a1* KO fed a chow diet tended to have a lower body weight without changes in physical activity, food intake but with a significantly lower energy expenditure. Paradoxically, this study detected a reduction in energy expenditure despite evidence of an elevated energy expending pathway in the adipose tissue of *Sult1a1* KO mice.

Weight matched WT and *Sult1a1* KO mice were placed on a HFD and body weight was tracked for 13-weeks. Like CT humans, *Sult1a1* KO mice on a HFD are resistant to weight gain (7). The body composition measurements from the mice on a high fat diet indicated that at 14-weeks old, there was no difference in the lean mass between the genotypes but a statistically significant reduction in fat mass. However, reduction in fat mass appeared to be transient and at 20-weeks of age lean mass and fat mass is similar between the genotype. In this study we were not able to investigate this observation further however the results suggest that there may be some compensatory mechanisms that become active mid-way through the study period observed. In contrast, the transient effect of reduced fat mass observed in the Sult1a1 KO mice was not observed in a cohort of CT females on an overfeeding protocol. Body composition of the females was measured at baseline, at the end of the 30-day fat overfeeding protocol and 30 days after the fat overfeeding protocol ended and there was a persistent reduction in fat mass that lasted even after the study protocol ended. Future studies could investigate the effect of sex on the body composition to determine if Sult1a1 KO female mice also experience a transient reduction in fat mass.

The results of this study show that *Sult1a1* deletion had a very minor effect on the glucose homeostasis. On a high-fat diet, Sult1a1 mice were less hyperinsulinemic and future studies could investigate if this is protective against insulin resistance. Increased adipose tissue weight (35), immune cell infiltration and fibrosis of the adipose tissue are characteristic of insulin resistance (36, 37). These features were reduced/less present in the adipose tissue of *Sult1a1 KO* mice suggesting that over time the KO mice might be protected against insulin resistance. Due to limited availability of tissues, further studies to investigate glucose uptake ex vivo, insulin signaling and beta-cell function were not possible.

As a previous study described changes in mitochondrial respiration of CT adipose tissue (12), the respiratory capacity of the adipose tissue from *Sult1a1* KO was measured. Subcutaneous adipose tissue from Sult1a1 KO mice had a significant increase in leak respiration accompanied by an increase in expression of UCP1 and outer mitochondrial membrane protein VDAC. Together these observations indicate browning of the subcutaneous adipose tissue from *Sult1a1* KO mice. Subcutaneous adipose tissue biopsies from CT and control individuals had similar leak respiration. UCP1 was not detected in the adipose tissue samples collected from CT and control individuals. A possible reason adipose tissue browning was observed in the *Sult1a1* KO mice and not CT individuals could be due to differences in the capacity for adipose tissue browning of the anatomical sites examined. In mice, the iWAT (a subcutaneous depot) is most prone to adipose tissue browning in contrast to eWAT (a visceral depot) which has a lower propensity for browning. One study suggests that humans and mice have opposing patterns of adipose tissue browning which could explain differences observed in leak respiration and UCP1 expression (38). Future studies could aim to measure additional key thermogenic markers.

By studying the Sult1a1 KO mice, we aimed to understand the contribution of reduced Sult1a1 expression to the CT phenotype. This work associates reduced Sult1a1 expression with a tendency for lower body weight and transient leanness. Additionally, this study identifies a novel role for Sult1a1 in body weight regulation and adipose tissue browning.

One possible mechanism driving adipose tissue browning in *Sult1a1* KO mice is reduced catecholamine metabolism. Catecholamines, epinephrine and norepinephrine, are substrates for beta-adrenergic receptors which when activated recruit inducible beige adipocytes in WAT. SULT1A1 does have some affinity for catecholamines which may lead to reduced metabolism of catecholamines possibly driving the adipose tissue browning observed. Further studies which measure catecholamine levels or Western Blotting for downstream catecholamine signaling would provide valuable insight into the association between Sult1a1 KO and browning of iWAT.

The current study has a number of limitations that need to be addressed when interpreting the data. First, the mouse model used simulated loss of Sult1a1 throughout the whole body, whereas it is unknown if individuals with CT have a widespread reduction in SULT1A1. A previous investigation into the CT phenotype focused on muscle and reported no decrease in SULT1A1. However, given that SULT1A1 is not strongly expressed in muscle, this may not be a representative tissue for assessing overall SULT1A1 reduction in CT. To address the role of SULT1A1 in adipose tissue function, an adipose tissue specific model may provide a better evaluation if SULT1A1’s impact on adipose tissue biology. Loss of Sult1a1 was modeled in male mice. We hypothesized that Sult1a1 KO female mice would mirror the body weight phenotype of women with CT. Male mice were selected to understand if there are sex specific response to Sult1a1 reduction. Subsequent experiments should include female mice to verify the effect of Sult1a1 on body weight and adipose tissue function. This study did not investigate BAT, perform histology on the iWAT or fully quantify the images (adipocyte size and collagen deposition), future studies could include full characterizations of these depots. In the H & E stained images, though there is a tendency for increased cellularity in WT mice, a limitation of this analysis is that the total nuclei counts do not confirm the identity of the infiltrating cells as macrophages or other immune cells.

Taken together, we here identify SULT1A1 and several other genes that are associated with human leanness. In an animal model we demonstrate that full body Sult1a1 KO mice have a tendency for a lean phenotype and a slight resistance to diet induced obesity. We identified adipose tissue browning as mechanism to explain the lower body weight observed. These findings have could have important implications for our understanding of human leanness and factors that may protect against body weight gain which can be taken into consideration when developing novel treatments for individuals living with obesity.

## Data Availability

The datasets presented in this study can be found in online repositories. The names of the repository/repositories and accession number(s) can be found below: https://www.ncbi.nlm.nih.gov/geo/, GSE167231.

## Supplementary material

Supplementary Figure 1 Linear regression of EE and body weight; EE and RER light dark split. **(A)** Linear regression of energy expenditure and body weight of chow-fed mice at 14-weeks of age. There was homogeneity of the regression slopes as the interaction term was not statistically significant F (1, 23) = 3.276, p = 0.083. The Shapiro-Wilk test was not statistically significant indicating normality of the residuals (p = 0.422) and Levene’s test was not significant (p = 0.125) indicating homogeneity of the residual variances for both genotypes. **(B)** Linear regression of energy expenditure and body weight of HFD-fed mice at 14-weeks of age. There was homogeneity of the regression slopes as the interaction term was not statistically significant F (1, 24) = 0.335, p = 0.568. The Shapiro-Wilk test was not statistically significant indicating normality of the residuals (p = 0.140) and Levene’s test was not significant (p = 0.277) indicating homogeneity of the residual variances for both genotypes. **(C)** EE of chow and HFD-fed mice. Data displayed as the total average EE during the 24-hour recording and the RER split into the dark (12-hour) and light (12-hour) phases. A t test was performed to compare the genotypes at total, dark and light for both diets. **(D)** Respiratory exchange ratio (RER) of chow and HFD-fed mice. Data displayed as the total average RER during the 24-hour recording and the RER split into the dark (12-hour) and light (12-hour) phases.

Supplementary Figure 2 Glycaemia of Sult1a1 KO mice and WT mice on a high-fat diet (HFD). n = 14-15 per genotype. Data represented as mean ± SEM. p values were calculated by using an unpaired Student’s t test. Data from **(A–C)** was collected from mice on HFD for 9 weeks. Data from panel D was collected two weeks later (after 11 weeks on a HFD) from the same mice shown in **(A–C)**. **(A)** Comparison of fasting blood glucose of Sult1a1 KO and WT mice on HFD for 9 weeks. **(B)** Time course of blood glucose concentration during the glucose tolerance test (GTT). Glucose bolus dosed at 2 g/kg body weight and administered by intraperitoneal (ip) injection. AUC glucose from 0 to 120 minutes from the time course shown in **(B)**. **(C)** Plasma insulin concentration during the GTT shown in panel B. At baseline (fasting), Sult1a1 KO had significantly reduced plasma insulin (p = 0.05), bar chart show in main **Figure 3B**. AUC insulin from 0 to 30 minutes from the time course shown in **(C)**. **(D)** Time course of insulin tolerance test (ITT) from mice on a HFD for 11 weeks. Insulin was administered by ip injection at a dose of 1 IU/kg body weight. AUC glucose from 0 to 120 minutes from the time course shown in **(D)**.

Supplementary Figure 3 Respiratory capacity of intact eWAT adipocytes isolated from Sult1a1 KO and WT mice on a standard diet. Higher but not statistically significant increased respiratory capacity in adipocytes isolated from eWAT of Sult1a1 knockout mice (n = 3-4). Basal respiration was measured using pyruvate (5µM) and malate (5µM) as substrates. Leak respiration was measured by inhibiting ATP synthase with oligomycin (2µg/ml). Titration of FCCP (0.5 µM steps) was used to determine maximal respiration. Non-mitochondrial respiration was measured by adding antimycin A (2.5 µM) and subtracted from all other respiratory states. Oxygen consumption was normalized by µg of DNA per chamber. Data represented as mean ± SEM. p values were calculated by using an unpaired Student’s t test.

Supplementary Figure 4 Rel. mRNA expression of Sult1a1 in tissues collected from mice fed either a standard chow diet or a HFD for 13-weeks. Data represented as mean ± SD with 7-15 biological replicates/genotype/diet. p values were calculated by using an unpaired Student’s t test.
