# Supplementary material for: Loss of Sult1a1 reduces body weight and increases browning of white adipose tissue
Source: Front Endocrinol (Lausanne). 2024 Dec 4;15:1448107. doi: 10.3389/fendo.2024.1448107 (PMC11656314; doi:10.3389/fendo.2024.1448107)
Supplement: Supplementary Figure 1 — Linear regression of EE and body weight; EE and RER light dark split. (A) Linear regression of energy expenditure and body weight of chow-fed mice at 14-weeks of age. There was homogeneity of the regression slopes as the interaction term was not statistically significant F (1, 23) = 3.276, p = 0.083. The Shapiro-Wilk test was not statistically significant indicating normality of the residuals (p = 0.422) and Levene’s test was not significant (p = 0.125) indicating homogeneity of the residual variances for both genotypes. (B) Linear regression of energy expenditure and body weight of HFD-fed mice at 14-weeks of age. There was homogeneity of the regression slopes as the interaction term was not statistically significant F (1, 24) = 0.335, p = 0.568. The Shapiro-Wilk test was not statistically significant indicating normality of the residuals (p = 0.140) and Levene’s test was not significant (p = 0.277) indicating homogeneity of the residual variances for both genotypes. (C) EE of chow and HFD-fed mice. Data displayed as the total average EE during the 24-hour recording and the RER split into the dark (12-hour) and light (12-hour) phases. A t test was performed to compare the genotypes at total, dark and light for both diets. (D) Respiratory exchange ratio (RER) of chow and HFD-fed mice. Data displayed as the total average RER during the 24-hour recording and the RER split into the dark (12-hour) and light (12-hour) phases. [file DataSheet1.pdf]

Supplementary Materials for

Loss of Sult1a1 reduces body weight and increases browning of white adipose tissue

Springer et al.

Corresponding author: Jörg Hager, jorg.hager@rd.nestle.com

Front. Endocrinol. 15:1448107.
doi: 10.3389/fendo.2024.1448107

The PDF contains:

Supplementary figures S1 to S4

Fig. S1

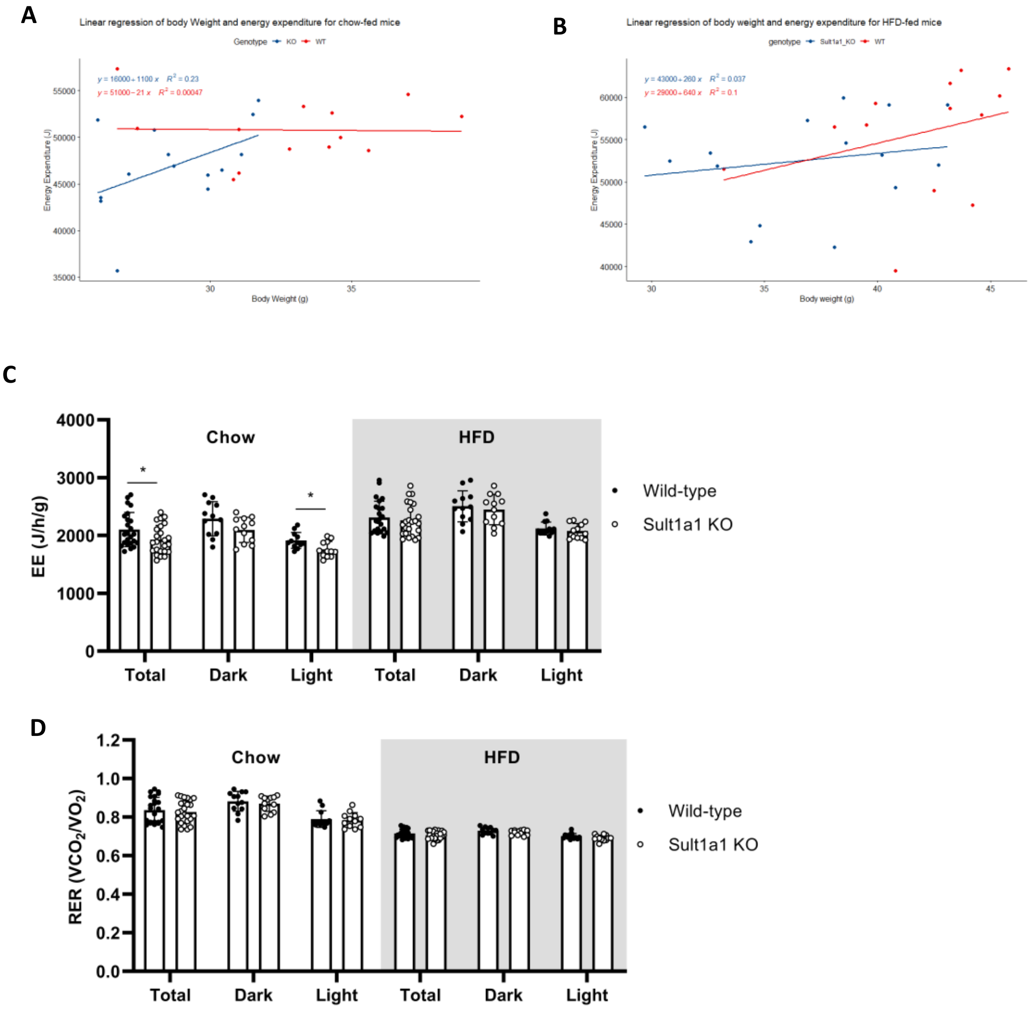

A
Linear regression of body Weight and energy expenditure for chow-fed mice
Genotype KO WT
y = 16000 + 1100 x R^2 = 0.23
y = 51000 − 21 x R^2 = 0.00047
Energy Expenditure (J)
55000
50000
45000
40000
35000
30
35
Body Weight (g)
B
Linear regression of body weight and energy expenditure for HFD-fed mice
genotype Sult1a1_KO WT
y = 43000 + 260 x R^2 = 0.037
y = 29000 + 640 x R^2 = 0.1
Energy Expenditure (J)
60000
55000
50000
45000
40000
30
35
40
45
Body weight (g)
C
4000
3000
2000
1000
0
EE (J/h/g)
Chow
HFD
*
*
Total
Dark
Light
Total
Dark
Light
Wild-type
Sult1a1 KO
D
1.2
1.0
0.8
0.6
0.4
0.2
0.0
RER (VCO2/VO2)
Chow
HFD
Total
Dark
Light
Total
Dark
Light
Wild-type
Sult1a1 KO

Fig. S2

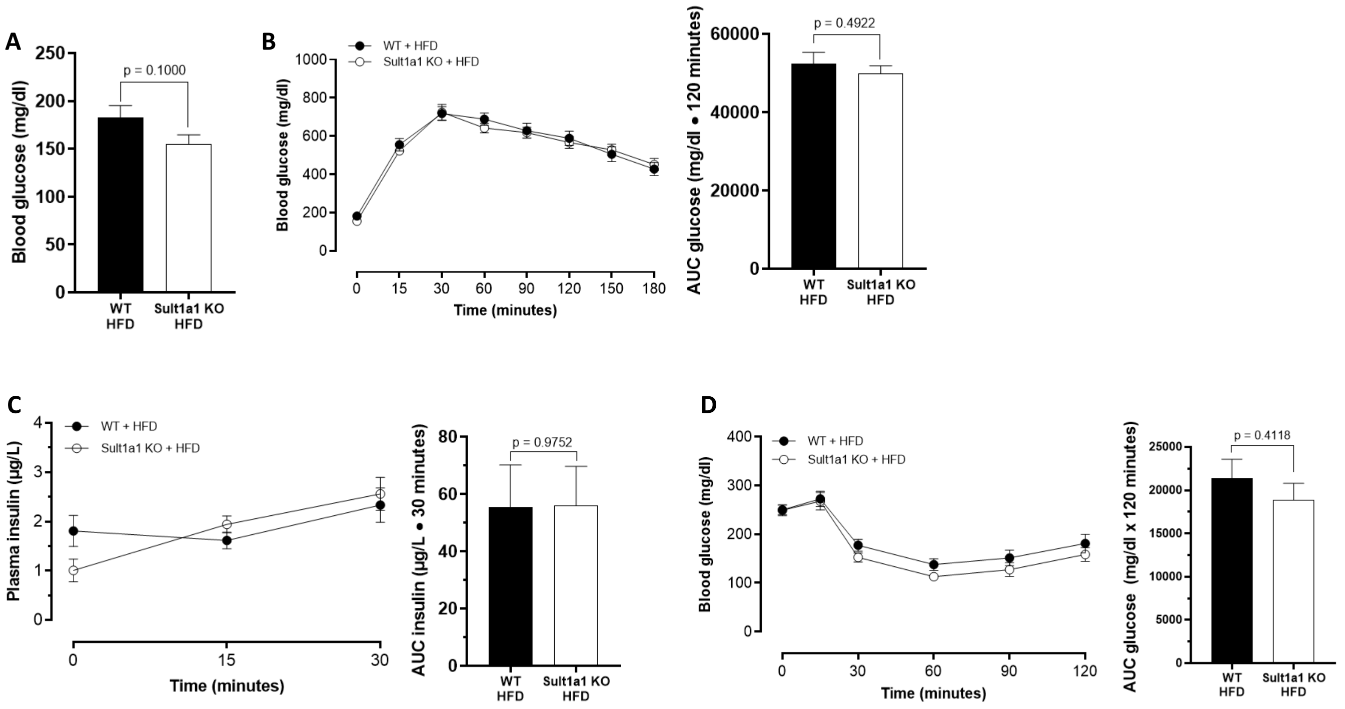
A
Blood glucose (mg/dl)
250
200
150
100
50
0
p = 0.1000
WT HFD
Sult1a1 KO HFD
B
WT + HFD
Sult1a1 KO + HFD
Blood glucose (mg/dl)
1000
800
600
400
200
0
0 15 30 60 90 120 150 180
Time (minutes)
AUC glucose (mg/dl • 120 minutes)
60000
40000
20000
0
p = 0.4922
WT HFD
Sult1a1 KO HFD
C
WT + HFD
Sult1a1 KO + HFD
Plasma insulin (µg/L)
4
3
2
1
0
0 15 30
Time (minutes)
AUC insulin (µg/L • 30 minutes)
80
60
40
20
0
p = 0.9752
WT HFD
Sult1a1 KO HFD
D
WT + HFD
Sult1a1 KO + HFD
Blood glucose (mg/dl)
400
300
200
100
0
0 30 60 90 120
Time (minutes)
AUC glucose (mg/dl x 120 minutes)
25000
20000
15000
10000
5000
0
p = 0.4118
WT HFD
Sult1a1 KO HFD

Fig. S3

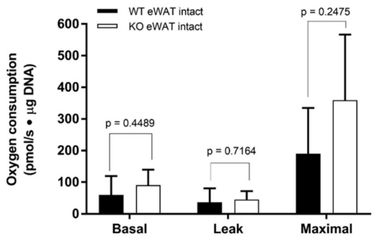

WT eWAT intact
KO eWAT intact
Oxygen consumption (pmol/s • µg DNA)
600
500
400
300
200
100
0
p = 0.4489
p = 0.7164
p = 0.2475
Basal
Leak
Maximal

Fig. S4

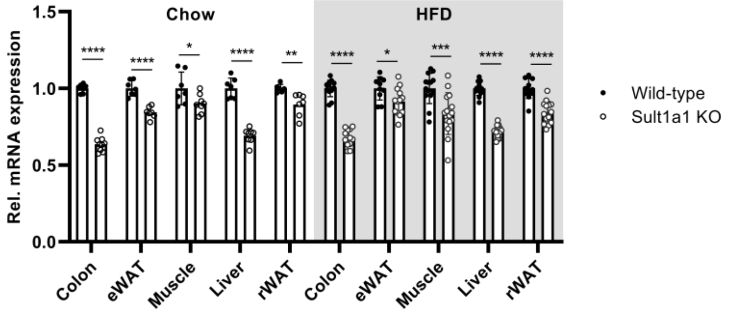

Rel. mRNA expression
1.5
1.0
0.5
0.0
Chow
HFD
****
****
*
****
**
****
*
***
****
****
Colon
eWAT
Muscle
Liver
rWAT
Colon
eWAT
Muscle
Liver
rWAT
Wild-type
Sult1a1 KO
